# Evidence that the woman’s ovarian cycle is driven by an internal circamonthly timing system

**DOI:** 10.1126/sciadv.adg9646

**Published:** 2024-04-10

**Authors:** René Ecochard, Joseph B. Stanford, Richard J. Fehring, Marie Schneider, Shahpar Najmabadi, Claude Gronfier

**Affiliations:** ^1^ Pôle de Santé Publique, Service de Biostatistique, Hospices Civils de Lyon, Lyon 69424 Cedex 03, France.; ^2^ Laboratoire Biostatistique Santé, Université Claude Bernard Lyon I, UMR CNRS 5558 UCBL, Lyon 69000, France.; ^3^ Office of Cooperative Reproductive Health, Division of Public Health, Department of Family and Preventive Medicine, University of Utah, Salt Lake City, 84108 UT, USA.; ^4^ College of Nursing, Marquette University, Milwaukee, P.O. Box 1881 WI, USA.; ^5^ Institute for Natural Family Planning, Milwaukee, P.O. Box 1881 WI, USA.; ^6^ Centre de Recherche en Neurosciences de Lyon (CRNL), Neurocampus, Inserm U1028, CNRS UMR5292, Université de Lyon, Lyon 69500, France.

## Abstract

The ovarian cycle has a well-established circa-monthly rhythm, but the mechanisms involved in its regularity are unknown. Is the rhythmicity driven by an endogenous clock-like timer or by other internal or external processes? Here, using two large epidemiological datasets (26,912 cycles from 2303 European women and 4786 cycles from 721 North American women), analyzed with time series and circular statistics, we find evidence that the rhythmic characteristics of the menstrual cycle are more likely to be explained by an endogenous clock-like driving mechanism than by any other internal or external process. We also show that the menstrual cycle is weakly but significantly influenced by the 29.5-day lunar cycle and that the phase alignment between the two cycles differs between the European and the North American populations. Given the need to find efficient treatments of subfertility in women, our results should be confirmed in larger populations, and chronobiological approaches to optimize the ovulatory cycle should be evaluated.

## Teaser

The menstrual cycle is driven by an internal clock and influenced by the moon cycle.

## INTRODUCTION

The ovarian cycle, also called the menstrual cycle, has a well-established circa-monthly rhythm in humans (*1*). The human menstrual cycle, which lasts an average of 29.3 days (*2*), is an infradian biological rhythm, because it is a recurrent biological process with a longer-than-24-hour period. However, is there an endogenous clock-like timer, external to the ovarian cycle itself, that determines its duration? Or, is the length of the menstrual cycle simply the sum of the times required for the pre- and postovulatory processes? There is a lack of good evidence to provide a definitive answer to this question. However, methods for numerical analysis of circadian rhythms (*3*) can be used to look for evidence that some sort of endogenous timing mechanism may be involved.

The length of the menstrual cycle of a woman at a given age varies around her personal average duration. Each typical ovulatory cycle consists of three parts or phases, and each phase is dedicated to the realization of a specific process. The first phase, the latency phase (*4*), begins on the first day of menstruation. The main process that takes place during this period is the selection of a dominant follicle that will later lead to ovulation. It lasts an average of 1 week, but its duration is more variable than the other phases of the cycle. Typically, the latency phase tends to shorten as the number of reserve follicles in the ovaries decreases as menopause approaches (*5*). The second phase, known as the fertile window (*6*), is less variable in duration. The processes of this phase are the growth of the dominant follicle until ovulation, as well as the secretion of fluid mucus in the cervix to receive spermatozoa. The ovulatory follicle grows from 10 to more than 20 mm in diameter over a relatively fixed period of about a week (*7*). If the first follicle fails to reach maturity, then another follicle takes over, extending the fertile window by several days. The third phase of the cycle, known as the postovulatory phase, lasts from the day after ovulation to the day before the next menstrual phase. It is considered to be fairly stable in duration, on average 12 to 13 days. However, there are variations (*8*), either physiological, due to the more or less rapid maturation of the corpus luteum (*9*), or pathological (*10*). Any change in the total length of the cycle is necessarily due to a change in one or more of these three processes.

There is some evidence to suggest that each of the three phases of the menstrual cycles may be under the influence of the circadian timing system (*11*, *12*). Disruption of circadian rhythms is associated with disturbances in menstrual function (*13*). Some authors have shown an effect of atmospheric pollution on the time of ovulation (*14*) or on the duration of the postovulatory phase (*15*). Others have described an effect of light exposure [see (*16*) for review] and shift work (*17*). However, to date, there is no strong or even moderate evidence to support the existence of an endogenous timing system that drives the entire menstrual cycle itself.

The observation of certain facts made it possible to emphasize the existence of the internal circadian clock. The first fact was the maintenance of a physiological rhythmicity of almost 24 hours, in individuals isolated from external influences (*18*). This was the first evidence for the existence of an internal circadian clock in humans. The existence of this clock was later confirmed, and its biological function was clarified (*19*). The period of this internal clock is close to 24 hours [24 hours and 9 min on average in healthy humans; (*20*)], and with a very narrow spread among individuals (SEM of 1 min). External environmental cues precisely reset and synchronizes the clock to the 24-hour day, with light being the most powerful synchronizer of the human circadian timing system. In the absence of a regular light-dark cycle, the circadian clock drifts on its own period (*21*, *22*), and the biological rhythms no longer exhibit 24-hour rhythmicity. Under certain conditions, both in animals and in humans, the phenomenon of relative coordination phenomenon can occur, with temporary episodes of synchronization between the circadian clock and its synchronizer (the light-dark cycle) and in-between episodes when the clock jumps from one phase to another or drifts (advances or delays) with a different circadian period (*23*).

In the case of menstrual cycles, if an endogenous clock is involved, then the same phenomena should occur, i.e., the cycle length should be highly stable within individuals and with a narrow spread across individuals (endogenous origin). Synchronizing mechanisms should exist to adapt the cycle (its phase) to a biological need that needs to be synchronized with external conditions, and abrupt changes in menstrual periodicity (a lengthening or shortening of the cycle) should be followed by rapid (jumps) or progressive (relative coordination) adjustment and recovery of the individual endogenous infradian period.

A non-endogenous mechanistic alternative for achieving a stable menstrual cycle, within individuals and across the population, could be one that relies on external environmental recurrent events. The theory that the duration of the menstrual cycle and human reproduction is closely related to the length of the lunar cycle has been frequently postulated (*24*–*27*). However, methodological weaknesses or statistical errors have generally called into question the results suggesting a significant relationship (*28*, *29*).

It has been suggested that some animals that use the high tides for reproduction have an internal clock of the same period as the lunar cycle (*30*, *31*) and that this internal clock would allow them to anticipate the tidal changes so that their reproductive processes can be synchronized with the tides. Recent results report that a single “circadian/circatidal” clock is sufficient to entrain behavioral patterns at lunar (tidal) frequencies in bivalves (*32*). With regard to the menstrual cycle, the existence of a relationship, even a weak one, between the processes of the menstrual cycle and the lunar cycle would indicate the probable existence of an internal timing system and a synchronizing effect of the lunar cycle. Recent work has confirmed the interest in this subject (*33*).

### Specific scope

**Fig. 1.**
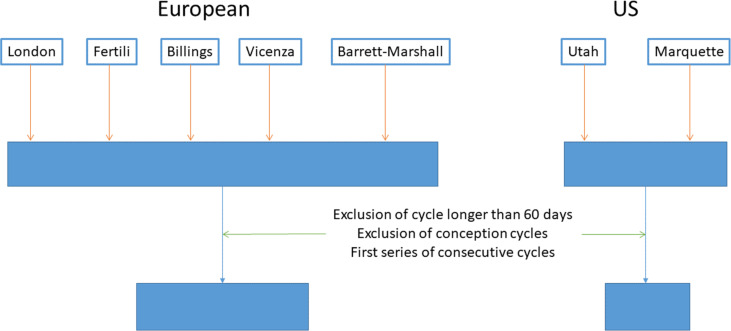
Composition of the European and North American datasets. Details are given in Table 1.

Here, we sought to gather evidence, if any, for an endogenous timing mechanism driving the menstrual cycle and for synchronization with the lunar cycle as a potential external driving mechanism. We used a large dataset of menstrual cycle parameters that were collected in studies conducted in Europe (*34*). To increase the level of evidence, the analyses were repeated on data collected in other studies conducted in North America (Fig. 1) (*35*, *36*). Statistical methods specifically designed to the study of rhythmic time series were applied to these two datasets. Our analysis focused, on the one hand, on the study of successive cycles (internal timing system) and, on the one other hand, on the relationship between menstruation and lunar phases (synchronizing role).

## RESULTS

The age of women in the European cohort at study entry was in the ranges 19 to 24 years, 25 to 34, 35 to 39, and 40 to 50 for 545 (24%), 1370 (59%), 263 (11%), and 125 (5%) women, respectively. In the North American cohort, the distribution in these age ranges was slightly different: 246 (35%), 393 (57%), 52 (7%), and 4 (<1%). Most women were <35 years old.

**Fig. 2.**
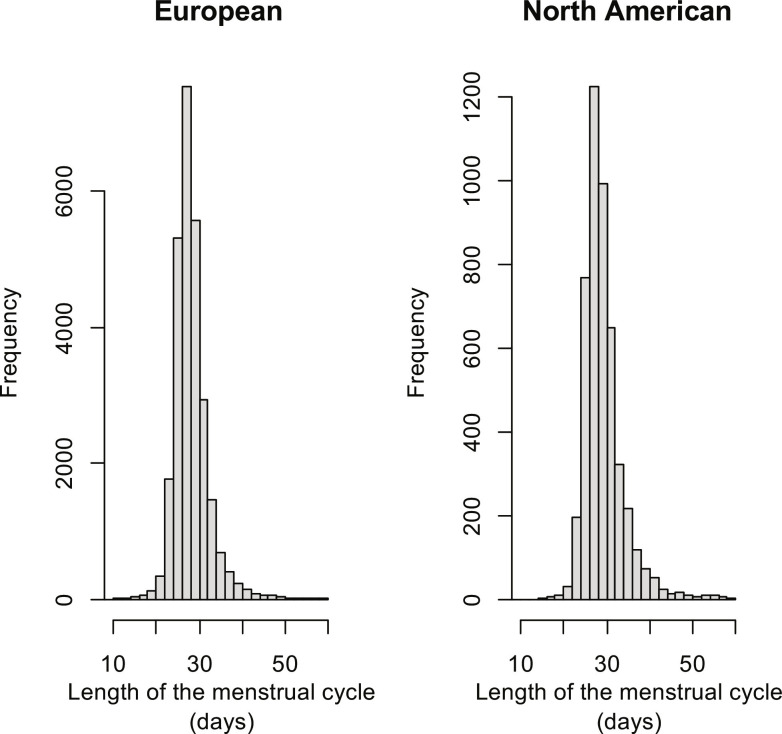
Distribution of menstrual cycle lengths.

Table 1 summarizes the number of cycles and women available for each analysis, with the associated selection criteria, stratified by data region. Figure 2 shows the distribution of cycle lengths in the two cohorts. For both the European and American populations, the distribution was slightly skewed to the right, with a mode slightly below 30 days. The first quartile, median, and third quartile of cycle lengths in the European cohort were 26, 28, and 30 days, respectively; those in the North American cohort were 27, 29, and 31 days, respectively.

**Table 1.** Datasets for each analysis.

Analysis	Selection criteria	Dataset	European cycles (women)	North American cycles (women)
		London	16,060 (1,057)	
		Fertili	5,199 (619)	
		Billings	1,970 (178)	
		Vicenza	1,911 (256)	
		Barrett-Marshall	1,772 (193)	
		Utah		3,137 (562)
		Marquette		1,649 (159)
			Total: 26,912 (2,303)	Total: 4,786 (721)
Actogram and description of oscillations	Series of 18 menstrual cycles or more		12,728 (425)	331 (12)
Correlation of the durations of successive cycles	Series of 13 menstrual cycles or more		16,568 (685)	–
	Series of seven menstrual cycles or more		–	3,529 (314)
Menstrual day and moon phase	Cycles whose first day date and duration are known		26,912 (2,303)	3,137 (562)

### Actograms

#### Global aspect

**Fig. 3.**
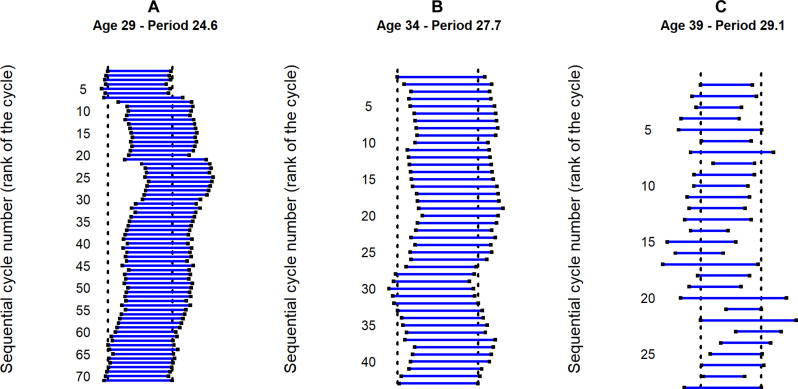
Examples of typical actograms for three European women. (**A**) Overall long-term stability with occasional jumps; (**B**) overall long-term stability with superimposed oscillations lasting less than a year; and (**C**) overall stability with rapid random fluctuations. The *x* axis represents the duration of each menstrual cycle, and the *y* axis the number of consecutive menstrual cycles. The length of each blue line represents the duration of individual menstrual cycles; each new cycle is shown below the previous one. For each woman, the mean cycle duration was used as an estimate of the infradian period of her menstrual cycle, and the actogram was plotted with a normalized time axis (modulo the infradian period). This makes it possible to visually follow how the duration of each menstrual cycle compares to the average cycle duration (black line), and how it varies over time. The reference time is, therefore, the average period of each woman’s menstrual cycle.

Observation of the actograms plotted with each woman’s cycle series reveals three characteristic patterns: (A) overall long-term stability with occasional jumps; (B) overall long-term stability with superimposed oscillations lasting less than a year (resembling relative coordination); and (C) overall stability with rapid random fluctuations. Figure 3 shows an example of each of these three types of actograms.

The actogram in Fig. 3A shows a stable menstrual rhythmicity from cycle 1 to 7, with a mean cycle length close to the average of 24.6 days. Then, we observe a shift (also known as a “phase jump” in circadian biology) to the right after the cycle 7, which is the result of a cycle that is much longer than the average cycle length. From the 8th to the 20th cycle, the actogram is stable again, with cycle durations close to the average cycle length. The actogram shows a second shift (phase jump) to the right after the 21st cycle, which is again a cycle that is much longer than the average cycle length. Then, the cycles show a long-term overall stability until the end (49 cycles), with a period shorter than the average cycle length. This first actogram highlights the need to take into account trends (drifts and jumps) whose duration is long, usually more than 1 year.

The actogram shown in Fig. 3B shows oscillations around a mean cycle length of 27.8 days. These oscillations take the form of a brief drift to the right when the cycles are slightly longer than average (menstrual cycle lengthening) and to the left when the cycles are shorter than average (menstrual cycle shortening). This second actogram shows oscillatory patterns that resemble relative coordination well-known in circadian biology and reveals oscillations of variable duration (period), often less than a year.

The actogram shown in Fig. 3C contains only rapid random fluctuations. This random fluctuation is superimposed on a mean cycle length of 29.1 days in this woman. The consecutive cycles are not similar in duration. Short and long cycles alternate in no particular order.

#### Description of oscillations

**Fig. 4.**
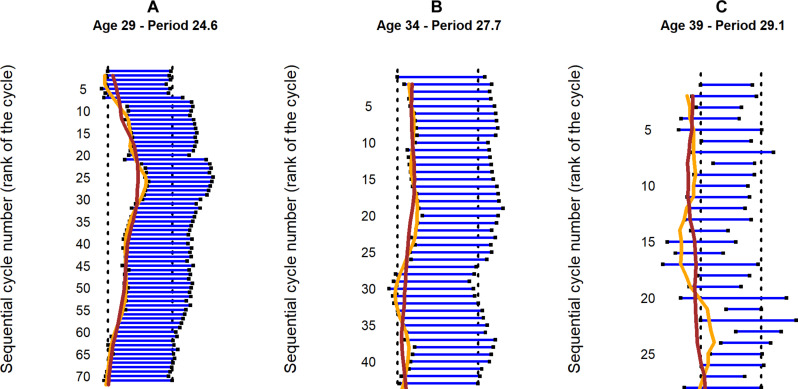
Examples of typical actograms of three European women, with oscillations estimated by smoothing. (**A**) Overall long-term stability with occasional jumps; (**B**) overall long-term stability with superimposed oscillations lasting less than a year; and (**C**) overall stability with rapid random fluctuations. The brown line shows the result of the smoothing using a long time period of the order of 1 year (12 cycles). The orange line shows the result of the fine Christiano-Fitzgerald smoothing, which emphasizes the oscillations.

We used smoothing techniques to quantitatively estimate the visually observed oscillations. In Fig. 4, the brown line shows the result of the smoothing over a period of nearly 1 year (12 menstrual cycles). The orange line shows the result of the fine Christiano-Fitzgerald smoothing, which emphasize the short-term oscillations. On the actogram in Fig. 4A, we see a wave before the first particularly long cycle (from cycle 1 to cycle 6) and a not very large (low amplitude) wave after this particularly long cycle (from cycle 10 to cycle 14). Another wave occurs after the second particularly long cycle: approximately from cycle 23 to cycle 28. On the actogram in Fig. 4B, three oscillations appear without being linked to cycles of particular duration. They occur respectively from the 7th to the 12th cycle, from the 14th to the 18th, and, more widely, from the 21st to the 26th cycle. The oscillations of the actogram (C) are both numerous and of large amplitude. However, they are quite different from those described in actograms (A) and (B). The oscillations of the actogram (C) are interspersed with smaller oscillations without any order. The oscillations of in actogram (C) are simply a reflection of a disordered variability in the length of the cycles.

Of the cycles of the 425 women in the European cohort, whose series is 18 menstrual cycles or more, 1846 oscillations of four cycles or more were observed, for a mean number of 4.3 oscillations per woman. Of these 1846 oscillations, 890 (48%) were observed in a stable state, 906 (49%) after an abrupt cycle lengthening (phase jump), and 50 (3%) were accompanied by fluctuations.

The 890 oscillations in a stable state were observed in 338 of 425 women (80%) in the European cohort. Of these, 150 oscillations of the 890 oscillations (17%) had an amplitude of 3 days or more. They were observed in 115 of 425 women (27%).

### Correlation of the durations of successive menstrual cycles

**Fig. 5.**
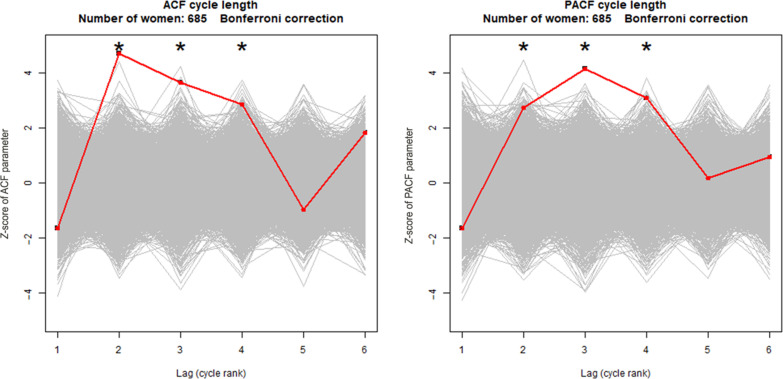
Correlations between the lengths of successive cycles (European dataset). The analysis is performed using the autocorrelation function (ACF; left) and the partial ACF (PACF; right). Each gray line is the average of the correlations obtained for each woman on randomly swapped data (permutation). The red line is the average of the correlations obtained on real non-swapped data. Significant correlations are indicated by asterisks (*) (bilateral test, with Bonferroni correction).

**Fig. 6.**
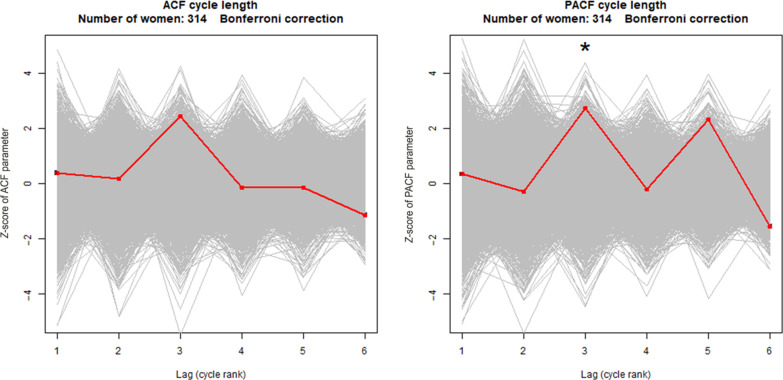
Correlations between the lengths of consecutive cycles (North American dataset). Analysis is performed using the ACF (left), and the PACF (right). Each gray line is the average of the correlations obtained for each woman on randomly swapped data. The red line is the average of the correlations obtained on real un-swapped data. Asterisk (*) indicates a significant correlation (bilateral test, with Bonferroni correction).

Figure 5 and Table 2 show the significance of the correlations between the lengths of successive cycles. The graph on the left shows the significance of the autocorrelation, and the graph on the right shows the significance of the partial autocorrelation. The null hypothesis of the absence of second, third, and fourth order autocorrelation was rejected (*P* < 0.0001, *P* = 0.0004, and *P* = 0.0024, respectively). The same applies the second, third, and fourth order partial autocorrelations (*P* = 0.0032, *P* < 0.0001, and *P* = 0.001, respectively). Only third-order autocorrelation (*P* = 0.0084) and third-order partial autocorrelation (*P* = 0.0030) were statistically significant for the North American dataset (see Fig. 6).

**Table 2.** Statistical significance of the correlation of the durations of successive cycles. ACF, autocorrelation function; PACF, partial ACF.

			Lag*					
Dataset	Cycles (women)	Model	1	2	3	4	5	6
European	16,569 (685)	ACF	0.9480	<0.0001	0.0004	0.0024	0.8306	0.0342
North American	3,529 (314)	ACF	0.3482	0.4354	0.0084	0.5582	0.5504	0.8718
European	16,569 (685)	PACF	0.9490	0.0032	<0.0001	0.0010	0.4268	0.1742
North American	3,529 (314)	PACF	0.3626	0.6072	0.0030	0.5806	0.0114	0.9364

*Consider the discrete sequence of menstrual cycle lengths. For lag 1, we compare the length of the menstrual cycle with the previous one. We calculate the correlation of the length of the menstrual cycle with the previous one. For lag 2, we calculate correlation with the second to last one. Etc. This table provides the *P* value. A significant *P* value indicates that the correlation is not compatible with simple chance.

### Menstrual day and lunar phase

To investigate the hypothesis that the menstrual cycle is synchronized by, or entrained to, the lunar cycle as an external synchronizer, we tested the relationships between the menstrual and lunar cycles.

In the European cohort as a whole, the first day of the menstrual cycle (menstrual day) occurred most frequently in the waxing crescent: 3533/26,912 (13.1%) (see Table 3). This percentage was only slightly higher than the average expected for an equal distribution among the eight lunar phases: 100/8 (12.5%). Nevertheless, both Pearson’s chi-square test (whose null hypothesis was the uniformity of the distribution of menstruation across the eight lunar phases) and the Rayleigh test were statistically significant. However, the Rayleigh test applied to the average per woman was not.

**Table 3.** Lunar phase of menstruation onset: Chi-square (χ^2^) and Rayleigh test.

	Subset	New	Waxing crescent	First quarter	Waxing gibbous	Full	Waning gibbous	Last quarter	Waning crescent	χ^2^ (*P* value)	Rayleigh test per cycle (*P* value)	Rayleigh test per woman (*P* value)
European	Total	3,362*	3,533	3,423	3,354	3,393	3,319	3,276	3,252	0.0214	0.0056	0.0986
North American	Total	356	367	382	422	431	382	407	390	0.0952	0.0226	0.0927
European	[28–30]	1,321	1,440	1,379	1,284	1,273	1,259	1,198	1,189	<0.0001	<0.0001	0.0090
North American	[28–30]	105	114	127	165	197	136	154	152	<0.0001	<0.0001	0.0313

*Number of menstrual cycles.

In the subset of women in the European cohort whose mean cycle length in the series was in the range [28–30], the first day of the menstrual cycle also occurred most frequently at the waxing crescent: 1440/10,343 (13.9%). The Pearson’s chi-square test and the Rayleigh test were statistically significant. The Rayleigh test applied to the average per woman was also statistically significant.

**Fig. 7.**
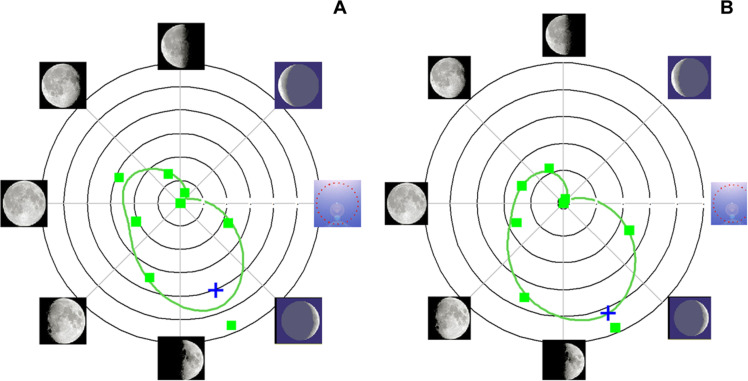
Menstrual day and moon phase (European dataset). In (**A**), the analysis includes all the data (26,912 cycles of 2303 women). In (**B**), the analysis focuses on the cycles of women whose average cycles are in the range [28–30] (9385 cycles of 775 women). The green squares represent the number of menstrual cycles (first day; see Table 3). The green line is the result of circular smoothing. The blue plus (+) sign indicates the significance on the bilateral test (Monte Carlo test with 5000 iterations), without Bonferroni correction. The circular *X* scale corresponds to the phase of the moon [clockwise: new moon (3 o’clock), waxing crescent, first quarter, waxing gibbous, full, waning gibbous, last quarter, waning crescent]. The *Y* scale corresponds to the frequency of occurrence of the first menstrual day as a function of the moon phase; the lowest frequency observed is in the center (respectively, 3252 on the left and 1189 on the right), and the highest is at the outermost edge (respectively, 3533 on the left and 1440 on the right). Moon images are adopted from (*84*).

**Fig. 8.**
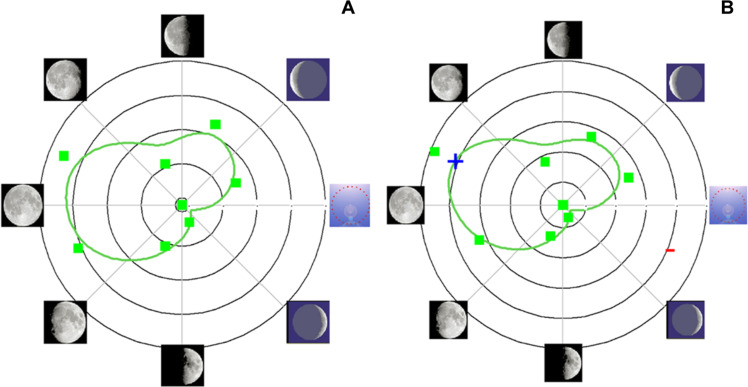
Menstrual day and lunar phase (North American dataset). In (**A**), the analysis includes all data for which menstruation start date was available (3137 cycles from 562 women). In (**B**), the analysis focuses on the cycles of women whose average cycles are in the range [28–30] days (1611 cycles from 222 women). The green squares represent the number of menstrual cycles that occurred at given lunar phases (first day; see Table 3). The green line is the result of circular smoothing. The blue plus (+) sign indicates the significance on the bilateral test (Monte Carlo test with 5000 iterations), without Bonferroni correction. The circular *X* scale corresponds to the phase of the moon [clockwise: new moon (3 o’clock), waxing crescent, first quarter, waxing gibbous, full, waning gibbous, last quarter, waning crescent]. The *Y* scale corresponds to the frequency of occurrence of the first menstrual day as a function of the moon phase; the lowest frequency observed is in the center (respectively, 356 on the left and 105 on the right), and the highest is at the outermost edge (respectively, 431 on the left and 197 on the right). Moon images are adopted from (*84*).

In the North American cohort, there was also a small but significant association between the onset of the menstrual cycle and the lunar cycle, but the menstrual cycle most often began at the full moon and not at the waxing crescent as in the European cohort. Only the Rayleigh test was statistically significant for the entire cohort. However, all three tests were statistically significant when applied to women whose average cycle length over the series was within the range [28–30]. Table 4 and Fig. 7 show the results of the Monte Carlo test for the European cohort. For the cohort as a whole, the first day of menses showed a significant association with the waxing crescent of the lunar cycle. In the sub-cohort of cycles of women whose average cycle length in the series was within the range [28–30], the higher frequency of menses starting at waxing crescent was confirmed. The same analysis on the North American cohort confirmed the significant association between menses and lunar cycle, the first day of the cycle starting at new moon (Fig. 8).

**Table 4.** Lunar phase of menstruation onset: Monte Carlo test (5000 iterations). Bold indicates more extreme value of each data set.

		Place of the observed value in the distribution of simulated values*							
	Subset	New	Waxing crescent	First quarter	Waxing gibbous	Full	Waning gibbous	Last quarter	Waning crescent
European	Total	0.4828	**0.9888**	0.7858	0.4180	0.6866	0.2592	0.1054	0.0726
North American	Total	0.0640	0.1390	0.3362	0.8778	**0.9600**	0.3468	0.6946	0.4454
European	[28–30]	0.6724	**0.9930**	0.9258	0.4334	0.3796	0.2944	0.0488	0.0372
North American	[28–30]	0.0158	0.0400	0.1770	0.8684	**0.9964**	0.3410	0.6954	0.6618

*The observed values are shown in Table 3.

## DISCUSSION

In this study, we looked for evidence of an internal timing mechanism driving the menstrual cycle, i.e., that would help determine the duration of the menstrual cycles. To do this, we looked for stability and instability of menstrual cycles, as well as evidence of jumps from one phase to another, and evidence of relative coordination around an intrinsic menstrual period (average cycle duration), as these phenomena are classically described in circadian biology. We also examined the relationships between the menstrual cycle and the lunar cycle. Our aim was to provide additional arguments in favor of (i) an endogenous timing mechanism regulating the length of the menstrual cycle, and (ii) an external synchronizing drive, at least in part, by the lunar cycle. Measurements of the partial or separate effects of the two possible causes (i) and (ii) are not available, as they would require the use of a complex statistical model and an even larger dataset than the one available to us.

Our findings include a correlation between the duration of successive menstrual cycles, and a statistically significant relationship between the menstrual cycle and the phases of the lunar rhythm. In what follows, we will discuss to what extent these two observed facts are arguments in favor of the existence of an internal timer (that expresses a near-monthly period) and an external synchronizer (the lunar cycle).

### Correlation between the duration of successive cycles

The raster plot graph (arrangement of the cycles on top of each other in the same graph) has been widely used in circadian biology (*37*). This study visually examines how the menstrual cycle dynamically varies around the mean cycle length (an estimator of the presumed circamonthly infradian period), as is done in the actogram, in two large populations of women, and on two continents.

We found three types of oscillations in the actograms. The first one (Fig. 3A) can be interpreted as a simple construction: After a particularly long cycle, the return to an average cycle length results in a wave on the actogram without particular significance. The phase jumps between one cycle and the next are preceded by a segment of stable oscillations (drifting at a given period) and followed by another segment of stable oscillations (drifting at the same or a different period than before the jump). This is reminiscent of what is observed in chronobiology when the circadian clock is barely synchronized to a particular environmental cycle (at the limit of the circadian clock’s range of entrainment) but, at some point, fails to stay synchronized (because it is outside the range of entrainment) and moves to another stable state (phase jumps) (*38*). The second type of oscillations that we have described (Fig. 3B), observed in a stable state, is important for the topic addressed here. It is the more or less long oscillations that reflect a correlation between the duration of successive cycles. It is the succession of several cycles slightly longer than the average cycle length that shows a wave to the right on the actogram, and, conversely, it is the succession of several cycles slightly shorter than the average that shows a wave to the left. These oscillations suggest that the duration of a cycle depends on the duration of the previous cycles. The third type (Fig. 3C), where the oscillations on the actogram are apparently random fluctuations in cycle length, can also be interpreted as having no particular significance.

To verify the significance of this correlation between successive cycle durations, we have estimated the statistical significance of the autocorrelation between successive cycle durations. There is an equivalence between a significant, albeit weak, autocorrelation between the duration of successive cycles and the oscillations on the actogram. We observed at least one wave in the steady state in the majority of the women in the European dataset who had a series of 18 or more consecutive cycles. The statistical significance of the existence of this autocorrelation between consecutive cycles was partially confirmed in the North American dataset. The correlation is sometimes positive and sometimes negative, but, on average, it is statistically very significantly different from zero.

### Partial autocorrelation across multiple cycles, mechanisms, and timing

The partial autocorrelation of ranks 2 to 4 was significant. Thus, the autocorrelation between the duration of a cycle and that of the four preceding cycles is not simply the reflection of a correlation between two consecutive cycles but a direct relationship between the duration of a cycle and that of the four preceding cycles. We advocate that there are two possible mechanisms for this.

The duration of follicular growth, from the primordial follicle to the ovulatory follicle, which is about 90 days, or three ovulatory cycles (*39*), can be translated into a visually stable menstrual cycle on the actograms. It is conceivable that the specific hormonal processes involved in the follicular cycle act on the growing passage and that this influences the length of the cycle in which one of them will end up ovulating. With such a homeostatic mechanism, which could be visualized as an hourglass process, we would see a relative stability of the menstrual cycle and occasional jumps with longer cycle durations when an ovulatory cycle fails before producing a mature follicle that triggers the initiation of a new follicular growth. However, this would not produce shorter cycles, as seen in the relative coordination oscillation that we described (Fig. 3, A and B), but only longer cycles (jumps to longer cycles and/or lengthening of the period of the menstrual cycle).

The other possible mechanism, in favor of the existence of a clock-like timer, is evoked by the oscillations of the actogram (Fig. 3B). The slow oscillations around the mean infradian period of the menstrual cycle are reminiscent of what is observed with a spring in action. The more it is stretched or compressed, the more it returns with force to its intrinsic length. This mechanism is also reminiscent of the relative coordination phenomenon described in circadian biology, which reflects both the existence of an endogenous clock and an acute effect of an external synchronizer on the circadian timing system (producing oscillations of phase around the endogenous period). Actually, the fact that the circadian clock can be mathematically modeled by a limit cycle that also explains relative coordination [or the rhythmic scalloping of the circadian phase; (*40*)] is a strong argument for a clock-like drive of the ovulatory cycle. For the menstrual cycle, this suggests the existence of a mechanism that corrects the difference between the current cycle length (current period of the rhythm) and that of the endogenous period of the menstrual cycle: A few shorter cycles would compensate for a series of a few longer cycles, so that the total cycle length would oscillate around the internal period of the menstrual cycle. In the same way that a few days are needed to compensate for a mismatch between the endogenous circadian phase and the day-night alternation after an intercontinental trip, several cycles may be needed to compensate for a discrepancy between the ovulatory cycle and an internal timer after one or more cycles of unusual length have occurred. We believe that the results of this work support such an endogenous clock-like mechanism, but further work is needed to test this hypothesis and unravel the mechanisms.

### Relationships with the lunar cycle

We also analyzed the relationship between the menstrual cycle and the phases of the moon. The close resemblance between the length of the lunar cycle and the length of the menstrual cycle has long raised the question of the relationship between these two phenomena. In the scientific literature, we have found publications that show the more frequent occurrence of menstruation after the new moon (*25*). Others, on the contrary, present data in favor of the occurrence of menstruation during the period when the moon is brighter or before and after the full moon (*24*, *26*, *41*). These contradictory conclusions, added to other articles that have not found any relationships, lead others to conclude that there is no link between the moon and menstruation (*29*, *42*). A recent article, referring to a text from 1806, took up the same assertion and claimed: “No correlation between lunar and menstrual cycles,” but the authors simply analyzed the menstrual cycles of a single woman to reach their conclusion (*43*).

In contrast, our analysis of two large and independent dataset suggests the possibility of a moderate but significant temporal association between the lunar cycle and the onset of the menstrual cycle. However, the menstrual cycle began more often at the waxing crescent in Europe, whereas it was at the full moon in North America. We do not have an explanation for this difference between the continents, but we believe that further study of this question is warranted. The differences observed between the European and American data may be due in part to differences in lifestyles between these continents, as well as changes in lifestyles between the time when the European data were collected and the much more recent collection of the American data. It should be noted that European data, collected in different countries and at different times (some European data were collected in the 1960s, others in the 1990s), are heterogeneous in terms of lifestyle. The emerging large databases, containing information on the cycles of several hundred thousand women, thanks to the smartphone applications used by women to record their menstruation, provide additional opportunities to address this question, which we hope will be carefully studied (*2*, *44*, *45*).

Three mechanisms have been proposed in the literature to explain the effects of the lunar cycle on living species, which occurs in various ways for other physiological processes in other animals.

The first is that of an acute effect of light on physiology. Some animals have a different activity according to the phases of the lunar cycle. Full moon nights are a period of vulnerability for some animals, as predators can reach them if they hunt or feed on these nights. In addition, light is known to alter the physiology of all mammals, including humans, even at low light levels (*46*–*48*), and its effect on the human menstrual cycle has been reported (*49*). The detrimental effects of “light pollution” and the existence of several pieces of work showing the effect of light on the menstrual cycle have led to the proposal that moon light could be responsible for the effect of the lunar cycle on biology (*16*). However, since the industrial era, very few Europeans and North Americans are likely to be exposed to alternating moonless nights and full moon nights, as nearly all are exposed to artificial light at night (*50*). Therefore, although we cannot formally exclude it, we believe that the direct effect of moonlight is unlikely to be involved in the synchronization between the lunar cycle and the menstrual cycle that we observe in our study.

The second mechanism invoked is that of the gravitational pull of the moon. In a recent article (*33*), the authors claimed that the gravitational forces of the moon “clearly contribute” to the onset of menses. We cannot completely rule out this mechanistic possibility; however, because the mass of an individual is very small, the gravitational pull of the moon is negligible and has never been unequivocally shown to have any physiological effects.

A third mechanism, based on an internal clock oscillating with a period that has evolved to be close to that of the lunar cycle, has been proposed to be involved in several biological organisms (*51*). These are organisms that live at the edge of the ocean and for which the tides are involved in controlling reproduction (*52*). As it will be discussed below, such a clock-like system may have been evolutionarily conserved in organisms that no longer live at the edge of the ocean. Whether this circalunar timing system relies on a specific circalunar clock machinery is unknown and cannot be argued on the basis of our descriptive data; however, given that circannual rhythmicities have been shown to depend on diurnal rhythmicity (*53*) and that tidal rhythms have recently been proposed to rely on a unique clockwork capable of generating both circatidal and circadian rhythms (*32*), it is conceivable that the circadian timing system is involved in circa-monthly rhythmicities.

In some species, a biological phenomenon related to reproduction can occur twice per lunar cycle, at the two phases when the tides are stronger, i.e., when the Sun, Earth, and Moon are aligned, at the new moon or at the full moon (*54*). Thus, the internal infradian timing system can have the same effect at two opposite times. A recent elegant study showed that the menstrual cycles of women with a period of more than 27 days were intermittently synchronized with the lunar cycle (*33*). The article shows series of menstrual cycles that alternate between synchronization with the new moon and synchronization with the full moon and also describes the relative coordination phenomenon that we see in our datasets and discussed earlier. Our work confirms and further extends both the oscillatory nature of the menstrual cycle and the possibly of synchronization at different phases in two larger datasets.

What could be the origin of this mechanism (*51*)? One of them, without having the means to prove it, could be related to a remnant of the evolution of species. It has been shown that biological clocks are of great importance in this embryonic development (*55*). Our aquatic ancestors gradually came out of the water and may have acquired an internal clock with a period close to that of the lunar cycle at the time when they were confronted with the tides. Then, during the millions of years of hominid evolution, this rhythm may have been active, possibly associated with the lunar night-light cycle. This may have allowed a relative synchronization of the cycles of women living together. It is known that, in some species, this synchronization can be favorable for the evolution of the species (*56*). Was this the case for hominids? This question has been debated (*57*, *58*).

What benefits might be derived from knowing that an internal clock drives the length of the menstrual cycle? This question may be related to the emerging interest in personalized therapeutics and circadian medicine (*59*). If the existence of an internal clock that controls the menstrual cycle is confirmed in further studies, then the medical treatment of ovulation disorders could use the chronobiological approaches that have proven successful in the treatment of cancer (*60*), sleep and circadian disorders (*61*), and depression (*62*). As one of the potential approaches, chronotherapy and/or bright light therapy could be tested in clinical trials to test their effect on the menstrual cycle and fertility (*63*, *64*).

In conclusion, we have noted several clues in favor of the existence of an internal timing system acting on the length of the menstrual cycle. The phase jumps, relative co-ordination, and partial autocorrelation observed in the menstrual cycle duration of both European and American cohorts support the existence of a cycle duration regulation with “memory” of the duration of previous cycles, which we interpret as best explained by a circa-monthly timing system of endogenous origin. The identified links between the lunar and menstrual cycles, although weak and inconsistent in terms of phase relationship between the European and North American cohorts, may lead to the periodicity of menstruation and, thus, ovulation, as a phenomenon possibly directly influenced by the lunar cycle or as a remnant of the evolutionary past. It can be treated as additional evidence for an internal near-monthly timing system with the lunar cycle as an external weak synchronizer.

Further research into the genetic regulation of the menstrual cycle will be important in understanding the chronobiology of the menstrual cycle. A variant of a gene involved in the regulation of follicular stimulating hormone was found to be associated with a change in cycle length (*65*). This shows that cycle length is genetically determined at some molecular level, but does not explain how the length of cycles could depend on the length of previous cycles, nor does it show how an internal timing system could affect the length of the menstrual cycle. Clinical factors are also known to correlate with cycle length, including age, parity, and age at menarche (*66*).

Given the need to find effective treatments for subfertility in women, our results should be confirmed in larger populations and the mechanisms deciphered from both human and animal studies. It would then be legitimate to study chronobiological approaches to optimize the ovulatory cycle, either by optimizing its stability or by improving its success. Such time-dependent therapeutic approaches have proven successful in many fields and could also prove effective here.

## MATERIALS AND METHODS

### Datasets

#### European dataset

The European dataset used in this study was compiled by the Department of Statistics of Padova (Italy) under the direction of Bernardo Colombo (*67*–*71*). This database contains information on 51,013 cycles from 3296 women from Great Britain, Italy, France, Germany, and Belgium and includes five sets of data, as shown in Fig. 1. The database has undergone consistency checks and has been used for several studies on the menstrual cycle (*72*, *73*), fecundity (*71*, *74*–*76*), and the evolution of the menstrual cycle with age (*77*, *78*). The data were obtained from women with no known cycle disorders.

In the database, the cycles were classified in order of occurrence and grouped into series of consecutive cycles. The same woman could participate in several series, interrupted by pregnancy or temporary interruption of data collection for other reasons. The outcome of the cycle, including the possible occurrence of pregnancy, was recorded. All these cycles were natural, without treatment with hormonal contraceptives or ovarian stimulation.

For this study, we have kept only the series of cycles for each woman between 20 and 50 years of age, whose first cycle was recorded between 1960 and 1997 inclusive. The series were stopped with the cycle preceding the pregnancy cycle or with other events that resulted in a cycle longer than 60 days. Only the first series of consecutive cycles for each woman was lastly included.

The European dataset, merged into a single file, includes 26,912 cycles from 2303 women for this study. Details of the number of women and cycles per dataset are given in Table 1.

#### North American dataset

The North American datasets were used for studies of female participants with no known health conditions that would cause cycle abnormalities. The first cohort was recruited from April 2008 to December 2010. Inclusion criteria for female partners of couple participants were that they had to be between 18 and 42 years of age, have a reported menstrual cycle of 21 to 42 days, have no history of hormonal contraceptive use in the past 3 months and, if post-breastfeeding, have experienced at least three cycles after weaning. More details about this cohort can be found in the original publication (*35*). Only one series of consecutive cycles were included. The series was stopped with the cycle preceding a pregnancy or any other event that resulted in a cycle longer than 60 days. This dataset, designated Marquette on in Fig. 1, contains one series of consecutive menstrual cycles each for 159 women (total, 1649 cycles).

The second cohort includes the menstrual cycles of women in the United States and Canada with no known health conditions that would cause cycle abnormalities who were enrolled in three different cohort studies (1990 to 2013). Eligibility criteria in the original studies included women 18 to 40 years of age, not pregnant at entry, having regular menstrual bleeding, and not breastfeeding or, if breastfeeding, not exclusively doing so. More details about this cohort can be found in the original publication (*36*). The series was stopped with the cycle preceding a pregnancy or any other event resulting in a cycle longer than 60 days. This dataset, designated Utah in Fig. 1, contains one series of consecutive menstrual cycles each for 562 women (total, 3137 cycles). Thus, the North American dataset includes 4786 (1649 + 3137) cycles from 721 (159 + 562) women.

#### Ethics

All datasets were obtained from studies that adhered to the tenets of the Declaration of Helsinki, and participants provided their informed consent to the use of their data. The European dataset is publicly available (*34*), and all necessary approvals were obtained from the University of Padova. The Utah dataset was collected in a study approved by the University of Utah Institutional Review Board (IRB) and local site IRBs. The dataset designated Marquette was collected in a study registered in the Clinical Trial Database under no. NCT00843336 and that received IRB approval through the University Office of Research Compliance of Marquette University.

### Factors studied and their availability in the datasets

Our unit of analysis was the menstrual cycle, with constant fluctuation of duration over time. We considered the woman’s self-reported first day of each menstrual bleeding as the first day of the cycle and the cycle time marker. Each cycle lasted up to and including the day before the first day of the next menstrual cycle, which was used to calculate the cycle length. Our outcomes of interest included periodicity and oscillations in the menstrual cycle length, autocorrelations of successive menstrual cycle lengths, and correlations of the first day of menses with the lunar cycle.

### Statistical analysis

We applied procedures classically used to analyze circadian rhythms. This analysis included the creation of actograms, time series analysis methods, and circular statistics.

#### Actograms

We created an actogram (also called a raster plot) for each woman. It consists of a series of superimposed lines, each line representing the length of a single menstrual cycle, and each new cycle is represented by a line below that of the previous cycle (Fig. 1). Note that we have named these raster plots “actograms” by analogy between the cycles of physical activity and the cycles of ovulatory/menstrual activity.

We chose to create actograms normalized to the average cycle length of each woman, as an estimate of the infradian period (modulo the menstrual cycle’s period). This allows each menstrual cycle be visually compared to the average length of a woman’s cycle. This average is considered her reference menstrual cycle’s period (analogous to the 24-hour duration on actograms used in the study of circadian rhythms). If one menstrual cycle is shorter, then the next one starts before the reference period. If one cycle is longer, then it is the other way around: The next one starts later in the reference period. As is usually the case with actograms, the advance or delay with respect to the reference period is made visible by a shift of the same duration. Mathematically, this is obtained by calculating the cumulative sum (cumsum) of the differences between the duration of each cycle and the duration of the reference period, i.e., the average duration of menstrual cycles.

The actogram is a graphical representation of a time series. In time series analysis, it is common to distinguish between drifts, called trends, and oscillations, also called wavelets. Each wavelet has a duration and an amplitude. To measure these durations and amplitudes in the menstrual cycles, we used a smoothing over a period of nearly 1 year (12 cycles), by a kernel smoothing method (*79*), and then we used a Christiano-Fitzgerald–type filter to estimate the wavelets (*80*). Calculation of the difference between these two smoothings, one global over a period of the order of a year and the other local to follow the wavelets consecutively, gives the length and the amplitude of each wavelet.

Wavelet durations and amplitudes were calculated for all women with a series of 18 cycles or more. Shorter series are not sufficient for correct estimation. Only oscillations with four or more cycles were considered for further analysis, and shorter ones were considered irregularities rather than oscillations.

#### Time series analysis

To quantify the similarity between the duration of consecutive cycles of the same woman, we first calculated the correlation between each cycle length and the six previous cycles. To do this, we used an autocorrelation function (ACF) (*81*). The ACF is a function that gives us values of the autocorrelation of any series with its lagged values. Part of the correlation between two cycles separated by a few months may be direct, and the other part may be from one cycle to the next. The ACF does not distinguish between these two types of relationship. Thus, to quantify the direct correlation between two cycles separated by one or more cycles, we used a partial ACF (PACF). This means that a partial autocorrelation of, for example, ranks 2 to 4 would be in favor of a direct relationship between the duration of a cycle and the duration of the preceding two to four cycles. Here, direct means that it is not the effect of a change in the length of the intermediate cycles.

The order in a temporal autocorrelation is defined as the distance between the two times at which the correlation is calculated. Thus, a second-order autocorrelation is the correlation between two cycles separated by one cycle, while a first-order autocorrelation is the correlation between two consecutive cycles. A second or third order autocorrelation may be statistically significant without the lower order autocorrelation being significant.

We applied these ACF and PACF functions to the series of consecutive cycles of all women in the European dataset whose series were at least 13 cycles long (i.e., 1 year or more). We retained all series of at least seven cycles for the North American database because we had shorter series for this database. However, these shorter series give less precise estimates.

We used a Monte Carlo permutation method to test for the existence of a relationship between successive cycles: The observed autocorrelation was compared with that obtained by swapping the cycles of each woman (*82*). This makes it possible to relate the observed correlation to the correlation calculated on the same cycles but arranged in other order. This was repeated 5000 times. Then, the average autocorrelation observed on all the women was compared to these 5000 average autocorrelations calculated on swapped data. The position of the observed value among the 5000 simulated values gave the *P* value, which can be interpreted as the probability that an observed autocorrelation between the cycles for each woman occurred by random chance alone.

We then plotted a graph showing the average correlations on swapped data and the observed average correlations. For comparability of results, we used the *z*-score as a unit: A deviation from the mean of more than 2 SDs reflects a threshold for statistical significance.

#### Circular statistics

The eight lunar phases that we considered were new moon, waxing crescent, first quarter, waxing gibbous, full, waning gibbous, last quarter, and waning crescent. These eight phases can be considered as categories, and, therefore, a Pearson’s chi-square was used to test the heterogeneity of the occurrence of the beginning of the menstrual cycle compared to the lunar cycle.

Furthermore, a circular statistics test that takes into account the order of appearance of these eight lunar phases will generally be more powerful to reject the null hypothesis of no association between lunar phase and the date of the first day of the menstrual cycle. We used the circular statistics test called Rayleigh test. It was first applied on all cycles, without taking into account the statistical nonindependence between cycles of the same woman. In a second step, we took into account the clustering of cycles for each woman. To do this, we first made a circular average of the lunar phases of occurrence of first day of menstruation of each woman and then applied the Rayleigh test to these averages. Then, we used the Monte Carlo method again, following the method proposed for the data grouped by the circular method (in our case, the cycles grouped by woman) (*83*). For the cycles of each woman, the simulated data were generated as follows: To the date of the first day of the first cycle of the series, a number randomly selected between 0 and 29 (days) was added. This places the beginning of the first cycle at any point in the lunar cycle. The observed duration of the cycles was then kept unchanged. This was done for each woman in the sample. This process was repeated 5000 times, resulting in many simulated samples. For each of them, the total number of cycles starting at each of the eight phases of the lunar cycle was calculated. Last, the observed frequency was compared to the 5000 simulated frequencies to calculate the *P* value for each of the eight phases of the lunar cycle. This *P* value can be interpreted as the probability of no association between the first day of menstruation and the lunar cycle.

In a sensitivity analysis for the circular statistics, the data were restricted to cycles of 28 to 30 days to more likely correlate with the lunar cycle. The observed data are presented in the form of a pie chart. To facilitate reading, an image of each lunar phase has been placed on the graph with some modification of the source data (*84*).

All calculations were performed using the R software (R version 4.0.3, The R Foundation for Statistical Computing). The significance level we used was 0.05. For bilateral tests with two alternative hypotheses, the threshold was 0.025. Unless otherwise stated, the Bonferroni correction was applied to all tests.
